# Physicians with self-diagnosed gadolinium deposition disease: a case series

**DOI:** 10.1590/0100-3984.2020.0073

**Published:** 2021

**Authors:** Richard C. Semelka, Miguel Ramalho

**Affiliations:** 1 Consulting PLLC, Chapel Hill, NC, USA.; 2 Department of Radiology, Hospital Garcia de Orta, EPE, Almada, Portugal.

**Keywords:** Gadolinium/adverse effects, Gadolinium DTPA/adverse effects, Physicians, Contrast media/adverse effects, Gadolínio/efeitos adversos, Gadolínio DTPA/efeitos adversos, Médicos, Meios de contraste/efeitos adversos

## Abstract

**Objective:**

The objective of this study was to allow physicians with self-diagnosed gadolinium deposition disease symptoms to report their own experience.

**Materials and Methods:**

Nine physicians (seven females), with a mean age of 50.5 ± 8.3 years, participated in this case series. Nationalities were American (n = 6), British, Portuguese, and Romanian. Medical practices included internal medicine (n = 2), trauma surgery, ophthalmology, gastroenterology, psychiatry, family medicine, obstetrics/gynecology, and general practice.

**Results:**

Genetically, eight of the physicians were of central European origin. Underlying autoimmune conditions were present in four. Symptoms developed after a single injection in one physician and after multiple injections in eight. The precipitating agent was gadobenate dimeglumine in four physicians, gadobutrol in three, gadoterate meglumine in one, and gadopentetate dimeglumine in one. The most consistent symptoms were a burning sensation, brain fog, fatigue, distal paresthesia, fasciculations, headache, and insomnia. Eight of the physicians were compelled to change their practice of medicine.

**Conclusion:**

In the various physicians, gadolinium deposition disease showed common features and had a substantial impact on daily activity. Physicians are educated reporters on disease, so their personal descriptions should spark interest in further research.

## Abstract (pt)

**Objetivo:**

O objetivo deste estudo foi possibilitar que médicos com sintomas de doença de deposição de gadolínio autodiagnosticada relatassem sua própria experiência.

**Materiais e Métodos:**

Nove médicos (sete mulheres), com média de idade de 50,5 ± 8,3 anos, participaram desta série de casos. As nacionalidades foram americana (n = 6), britânica, portuguesa e romena. As práticas médicas incluíram medicina interna (n = 2), traumatologia, oftalmologia, gastroenterologia, psiquiatria, medicina de família, ginecologia/obstetrícia e clínica geral.

**Resultados:**

Geneticamente, oito dos médicos tinham origem europeia central. Condições autoimunes subjacentes estavam presentes em quatro médicos. Os sintomas se desenvolveram após uma única injeção em um médico e após várias injeções em oito. O agente precipitante foi gadobenato dimeglumina em quatro médicos, gadobutrol em três, gadoterato meglumina em um e gadopentetato dimeglumina em um. Os sintomas mais consistentes foram sensação de queimação, confusão mental, fadiga, parestesia distal, fasciculações, cefaleia e insônia. Oito dos médicos foram forçados a alterar a sua prática médica.

**Conclusão:**

Em vários médicos, a doença de deposição de gadolínio mostrou características comuns e teve um impacto substancial na atividade diária. Os médicos são repórteres treinados sobre doenças, assim, suas descrições pessoais devem despertar interesse em pesquisas futuras.

## INTRODUCTION

It has long been known that gadolinium is retained in various tissues after the administration of either of the two classes of gadolinium-based contrast agents (GBCAs) in humans^(1-5)^ and animals^(6,7)^, reflecting the higher tissue concentration associated with the use of linear agents. There is a lack of histological evidence of cytotoxic effects of gadolinium deposits^(4,5)^.

Gadolinium deposition disease (GDD) is a newly described entity^(8-10)^. The term GDD has been proposed to describe a condition in which a patient with normal kidney function develops long-lasting symptoms after GBCA exposure, assuming that alternative conditions and causes have been excluded^(8-10)^. This entity has been met with skepticism among physicians, mainly because previous papers describing the disease have been criticized for flaws in their methodology^(11)^. In addition, because such patients have normal or near-normal renal function, they do not conform to the well-accepted understanding that only patients with advanced renal failure are at risk of developing long-standing disease, which in those with renal failure would be nephrogenic systemic fibrosis (NSF) secondary to GBCA administration^(12,13)^. Such patients typically develop NSF after being injected with linear agents that are less stable, especially gadodiamide, gadoversetamide, and gadopentetate dimeglumine^(13)^. Another troubling issue is that macrocyclic agents have also been reported to cause GDD^(8-10,14)^. Controversy also stems from the lack of broadly reported objective examination, laboratory, and imaging studies on these subjects. Physicians reporting their own experience with their self-diagnosed GDD may help promote further research into this condition.

## MATERIALS AND METHODS

All participants contacted the primary author of their own volition, based on the author’s experience with gadolinium retention and toxicity, which formed the basis of entry into this report. Self-reports were gathered between August 2016 and March 2020. The participants were prospectively given a template to follow. To establish the diagnosis of GDD, the participant physicians used early proposed criteria for the disease, which include the following:

- Patients should have undergone an imaging study involving the use of a GBCA in the last month prior to symptom onset.
- The symptoms should be new to the patient (i.e., not reflecting any pre-existing disease or symptoms observed before GBCA administration). Some symptoms include brain fog; pins and needles sensations; glove and stocking distribution of symptomatology (skin discoloration, pain, and subcutaneous tissue thickening); bone pain; and a burning sensation.
- Whenever possible, evidence should be obtained that gadolinium remained in their system for more than 30 days after administration.
- Patients should have had normal or near-normal renal function at the time of GBCA administration.

Nine physicians (seven females), with a mean age of 50.5 ± 8.3 years (range, 40-62 years), reported their disease history, including a brief personal description (age, gender, race, medical specialty, and medical history), the precipitating GBCA, symptoms, investigation, treatment, work status, and legal action.

Of the nine participating physicians, six were American, one was British, one was Portuguese, and one was Romanian. Two of the physicians worked in the field of internal medicine, whereas one each worked in the fields of trauma surgery, ophthalmology, gastroenterology, psychiatry, family medicine, obstetrics and gynecology, and general practice.

In all cases, the GDD was self-diagnosed. All of the affected physicians consulted other physicians and underwent extensive investigation to rule out multiple sclerosis and a comprehensive list of new autoimmune, malignant, and infectious diseases. All of the tests were negative.

## RESULTS

Data related to the affected physicians are summarized in Table 1. The disease originated 2 to 13 years before this report. The estimated glomerular filtration rate (eGFR) was calculated in all cases. The eGFR was calculated on the basis of the serum creatinine level, age, body mass index, and gender. The abbreviated Modification of Diet in Renal Disease formula was employed: $186x{\left(\mathrm{creatinine}/88.4\right)}^{1.154}x{\left(\mathrm{age}\right)}^{-0.203}x\left(0.742\;\mathrm{if}\;\mathrm{female}\right)x\left(1.210\;\mathrm{if}\;\mathrm{balck}\right)$. Renal function was normal (eGFR > 90 mL/min/1.73 m^2^) in eight subjects and near-normal (eGFR of 60-90 mL/min/1.73 m^2^) in one.

**Table 1:** Data related to physicians with self-diagnosed GDD.

Subject	Age (years)	Gender	Nationality	Specialty	Symptoms	Autoimmune history	Work status and legal action
1	40	Female	Portuguese	Psychiatry	Burning sensation and muscle pain; muscle stiffness and cramps; lower leg paresthesia; muscle fasciculations; fatigue; insomnia; transient difficulty in swallowing	Takayasu arteritis; ankylosing spondylitis	Discontinued work, no legal action
2	55	Female	American	Internal medicine	Bilateral pain extending from the forearms to the hands; burning sensation in a glove and stocking distribution and on the torso; fatigue; brain fog; muscle weakness	Autoimmune optic neuritis	Discontinued work, no legal action
3	40	Male	American	Ophthalmology	Burning sensation in a glove and stocking distribution; paresthesia of the extremities; sensory neuropathy of lower extremities; muscle fasciculations; insomnia; brain fog	None	Limited practice, contacted a personal injury attorney
4	59	Female	American	Family medicine	Muscle fasciculations; generalized burning sensation; blurred vision; tinnitus; headache; brain fog	None	Discontinued work, no legal action
5	62	Male	American	Gastroenterology	Fatigue; lower-extremity paresthesia	None	Works full time, no legal action
6	46	Female	Romanian	Obstetrics and gynecology	Transient mild laryngeal spasm; dull right rib pain; left leg paresthesia; blurred vision; whole- body paresthesia and electric shock sensation; burning sensation affecting the skin, with a pins and needles sensation affecting both forearms; fatigue/muscle weakness; insomnia; occasional headache; brain fog	None	Limited practice, now works full time, no legal action
7	44	Female	British	General practice	Pounding headache; headache; dysautonomia; arthralgia; brain fog; burning muscle pain; fatigue/muscle weakness	Ehlers-Danlos syndrome; celiac disease	Discontinued work, no legal action
8	57	Female	American	Internal medicine	Headache; lip numbness; leg cramps; burning sensation and pins and needles sensation in legs; myoclonic jerks; allodynia; fatigue; severe pain in the hands	Sarcoidosis	Limited practice, no legal action
9	52	Female	American	Trauma surgery	Right-sided burning sensation affecting the skin and subcutaneous tissue; insomnia; brain fog; headaches; gastroparesis; fasciculations; visual problems; glove and stocking distribution of pain, discoloration, and subcutaneous tissue doughiness	None	Discontinued work, no legal action

Genetically, eight of the physicians were of central European origin and one was half central European. Underlying autoimmune conditions were present in four physicians. Extensive testing for multiple diseases, including new (i.e., not pre-existing) autoimmune diseases, was conducted in all subjects, and all of the results were negative.

The disease appeared after a single injection in one physician and after multiple injections in eight. The precipitating agent was gadobenate dimeglumine in four cases and gadobutrol in three. The other precipitating agents were gadoterate meglumine and gadopentetate dimeglumine. It appears that all of the physicians who had multiple contrast injections received more than one type of GBCA.

The most consistent symptoms were a burning sensation (in all nine cases), brain fog (in six), fatigue (in six), distal paresthesia (in five), fasciculations (in four), headache (in five), and insomnia (in four). Eight of the physicians were compelled to change their practice of medicine: three stopped practicing medicine altogether; one stopped seeing patients but continued to practice administrative medicine; and four reduced the size of their practice (two of them considerably) and steered away from work that requires manual dexterity. Brain fog, a burning sensation, or both was the cause for ceasing to care for patients. At this writing, all of the physician-patients have used supplements, together with dietary modifications, and four had at least started specific treatment with DTPA chelation, which is ongoing in three. One physician consulted an attorney regarding the disease but did not pursue legal action. The others did not seek legal restitution. The timing of the symptom onset, number of GBCA doses, and clinical indications for GBCA-enhanced magnetic resonance imaging (MRI) are summarized in Table 2.

**Table 2:** Clinical indications for contrast-enhanced MRI, number of doses, precipitating dose, precipitating GBCA, and timing of the onset of GDD symptoms.

Subject	Clinical indication(s)	Number of doses	Precipitating dose (mmol/kg)	Precipitating GBCA	Timing of symptom onset after administration
1	Takayasu arteritis; ankylosing spondylitis	10	1.0	Gadoterate meglumine	Within hours
2	Possible multiple sclerosis (n = 5); concussion (n = 1)	6	0.6	Gadobenate dimeglumine	Within 2 weeks
3	Migraine headache with aura (n = 1); headaches/nausea (n = 1); posterior cervical lymphadenopathy (n = 1); firm, mobile skin nodule, anterior to the tibia, and leg pain (n = 1); abdominal MRI (n = 1)	5	0.2	Gadobenate dimeglumine	Within 1 day
4	Chronic hamstring tendon insertion avulsions (n = 1); leg pain (n = 2); MR arthrography; blurred vision, tinnitus, insomnia, and burning sensation (n = 1); MR angiography (n = 1)	5	0.1	Gadobutrol	Within days
5	Homonymous hemianopsia; moderate headache following intense exercise (n = 2)	2	0.2	Gadobenate dimeglumine	Within 2 weeks
6	Right-sided pulsatile tinnitus (n = 1); acute cholecystitis (n = 2)	3	0.2	Gadopentetate dimeglumine	Within days
7	Worsening of tinnitus (n = 3)	3	0.1	Gadobenate dimeglumine	Within hours
8	BRCA mutation (n = 4)	4	0.1	Gadobutrol	Within 3 days
9	Trauma after a motor vehicle accident (n = 1); symptoms that appeared after the first contrast-enhanced MRI (n = 11)	12	0.1	Gadobutrol	Within days

## DISCUSSION

In this study, all nine physicians with self-diagnosed GDD described their condition and experience. These physicians described the development of new symptoms after the administration of GBCAs, not reflecting a pre-existing disease (i.e., symptoms that they had not experienced before the GBCA administration). All of the physicians were initially under investigation for conditions that do not have symptoms similar to those described in GDD. Those conditions included Takayasu arteritis with ankylosing spondylitis, concussion, headaches/nausea, chronic hamstring tendon insertion avulsions, homonymous hemianopsia with moderate headache following intense exercise, acute cholecystitis, Ehlers-Danlos syndrome, BRCA mutation, and major injuries following a motor vehicle accident. Eight of the physicians had normal renal function at the time of the GBCA administration, and one had an eGFR in the 60-90 mL/min/1.73 m^2^ range, despite having previously been normal (> 90 mL/min/1.73 m^2^) and having no known risk factors for chronic kidney disease. All physicians also underwent extensive tests for multiple diseases, the results of which were all negative. Some had a pre-existing autoimmune disease, as described in the Results section.

In this era of increased patient input into medical research^(15,16)^, a novel aspect of the present study is that all of the reporting sufferers were themselves physicians. We are not aware of another study in which the patient population comprised only physicians.

In previous descriptions of GDD, a variety of symptoms have been reported. However, those most frequently described included symptoms that are also present in early NSF^(12,13)^, such as changes of the distal arms/hands and distal legs/feet in a distribution described as glove and stocking, although those symptoms are less severe in GDD. In addition, GDD presents with some symptoms that have not been widely reported in NSF^(8-10)^: brain fog; a new form of headache not previously experienced; blurred vision; dry eyes; a burning sensation (affecting the skin and subcutaneous tissue); intense bone or joint pain; and sharp pins and needles pain (neuralgia). In the present study, the self-reports of the physicians are in keeping with what has been previously reported on GDD^(8-10,14)^. The most common symptom (reported by all nine physicians) was a burning sensation, followed by brain fog, fatigue, distal paresthesia, headache, fasciculations, and insomnia. In addition, most (seven) of the physicians were female and all were of at least 50% white central European ancestry, which is also in agreement with what has been reported in other studies of GDD^(8-10)^.

One distinctive feature seen in these physician reports, contrary to what has been described in NSF, is that the disease arose following the administration of all types of GBCAs, regardless of their molecular structure, and not primarily from the least stable linear agents. In this case series, the precipitating agent was gadobenate dimeglumine in four of the physicians, gadobutrol in three, gadoterate meglumine in one, and gadopentetate dimeglumine in one. In this regard, the development of symptoms of GDD resembles an acute hypersensitivity reaction, because the symptoms can arise after the administration of any GBCA agent and often appear quite soon after GBCA administration, rather than months later^(14)^. Notably, underlying autoimmune conditions were present in four of the nine physicians.

An essential aspect of these reports is the debilitating nature of the disease reported. As previously mentioned, eight of the nine physicians were compelled to change their practice of medicine. It is noteworthy that the impetus for leaving or reducing the workload in medical practice was not driven by pain in most of the individuals, six of the physicians reporting that their main motivation was the brain fog, the burning sensation, or both.

Recent articles skeptical on GDD emphasized the concern that this entity may result in major medical malpractice lawsuits and stated that this may be the primary motivator of the apparent sufferers^(17,18)^. Nevertheless, in our sample, only one of the nine physicians sought legal assistance. Therefore, financial compensation did not appear a major motivator, despite the symptom severity and the disruption that the disease had caused in their lives. It is also noteworthy that many of the physicians evaluated in this report have maintained a low profile about their illness and were highly concerned about confidentiality. In fact, six physicians who contacted the primary author about their disease were not included in our sample. As part of the evaluation process, those physicians provided a full history of their condition but declined to participate in the study. We believe that their reluctance was primarily linked to concerns that there would be some form of retaliation or a negative impact on their position/employment.

On the basis of the current literature, one of the problems related to GDD is the difficulty in establishing a definitive diagnosis^(8,9)^, which is related to various factors: the apparent infrequency of the disease; the fact that it can present delayed manifestations; and the lack of a dose threshold for the development of symptoms, which may arise after one injection or after multiple injections. Because all of the individuals in this case series were physicians, we relied on their medical knowledge to establish an association between GBCA administration and the development of symptoms.

The information provided herein emphasizes the role of the physician, whose training should have honed their skill in listening to, as well as in observing the symptoms reported by, their patients. Despite the fact that the patients in our study were themselves physicians, they often did not make the association until after they had received multiple injections of GBCAs, each injection worsening their condition.

The authors hope that this report will enlighten physicians regarding GDD, even if they do not always recognize the disease. If a patient with normal renal function describes symptomatology that is “NSF-like” after a GBCA-enhanced MRI examination, we recommend that they not receive a GBCA in any future examinations.

Some authors have raised the question of whether GDD is a “real” disease, even speculating that it might be not only artifactual but also driven by litigation^(11,17)^. However, those objections are based on theory alone, with no apparent effort to examine patients with GDD and provide an alternate diagnosis. Parillo et al.^(19)^ found that the onset of new GDD-like symptoms were more common at 24 h after exposure to GBCA than at 24-h after unenhanced MRI. Among GDD-like symptoms, fatigue and mental confusion (brain fog) were the symptoms most often reported by the physicians in our sample. One recent paper^(20)^ presented a case of fibromyalgia that emerged after exposure to GBCAs and exacerbated after additional exposures, closely resembling the signs and symptoms described in GDD. Our opinion is that the authors of that paper were reporting a case of GDD and not fibromyalgia. Another recent study showed that cytokine profiles determined from serum samples differed between individuals with GDD and those in a group of age- and gender-matched controls^(21)^.

Our study has some limitations. The main limitation is the small sample size. As previously mentioned, additional physicians with self-diagnosed GDD who contacted the primary author did not choose to contribute their anonymized account. In addition, the fact that most of physician-patients contacted the principal author because they believed that they had the disease in question probably represents a selection bias, albeit an ostensibly unavoidable one. Another limitation is the subjectivity in the description of each clinical case. However, because all the patients were physicians, we attempted to maintain as much as reasonable their self-reported reflections on their self-diagnosed disorder, in order to preserve the authenticity of the expert testimony. Furthermore, not all of the participants knew which GBCA was administered in every examination. Nevertheless, they recognized the precipitating one. The general description is that with each subsequent GBCA injection, the symptoms worsened, and that the symptoms were often subtle following the earlier injections. This underscores the importance of recognizing GDD early in its course, given that subsequent GBCA injections invariably appeared to make the symptoms worse.

## CONCLUSION

Here, we have evaluated the self-reports of physicians with self-diagnosed GDD in which they describe their own experiences. The reported condition showed considerable consistency among different individuals. This case series also reveals the substantial impact that GDD may have on daily activity. We hope that this report will engender more interest in this line of research.
